# Segmental Arterial Mediolysis: A Multiguised Vasospastic Arteriopathy with Collateral Mesangial Cell Hyperplasia and Cardiac Toxicity Generated by Norepinephrine and Hyperdense Adrenoceptors Alone or by Crosstalk with Other Pressor Agents

**DOI:** 10.1155/2021/2046566

**Published:** 2021-11-23

**Authors:** Richard E. Slavin

**Affiliations:** 1793 Childs Rd, Lake Oswego, Oregon, USA

## Abstract

Segmental arterial mediolysis (SAM), an uncommon vasospastic arteriopathy occurring in the muscular arteries innervated by the peripheral sympathetic nervous system, usually presents with catastrophic abdominal and retroperitoneal hemorrhages in elderly patients. SAM is initiated by the coupling of norepinephrine to plastically derived hyperdense foci of alpha-1 adrenergic receptors on the sarcolemma of arterial muscle. This ligand is created by stimuli signaled by iatrogenic sympathomimetic agonists, some beta-2 agonists, or an excessive release of adrenal catecholamines. Coupling of this ligand with cytoplasmic heterotrimeric Gq protein excessively signals a cascade of biochemical events generating two principal lesions of injurious-phase SAM—the shearing of the outer media from the adventitia and an overload of cytoplasmic calcium ions toxic to mitochondria causing mediolysis and/or apoptosis. The massive hemorrhages are caused by ruptured gap aneurysms created by the transmedial loss of the medial muscle. A norepinephrine-directed reparative response rapidly develops either resolving angiographic injurious lesions or creating a body of vascular disorders, the new guises of SAM with ischemic clinical profiles. These present in the epicardial, vertebral, intestinal, and retroperitoneal arteries, often in younger females as fibromuscular dysplasia, dissecting hematomas, and persistent aneurysms. Norepinephrine can crosstalk with other pressor agents to create SAM lesions—serotonin with idiopathic pulmonary hypertension and persistent pulmonary hypertension in the newborn, histamine in spontaneous coronary artery dissections with eosinophilia, and endothelin-1 in a field effect generated by SAM that creates venous fibromuscular dysplasia. Norepinephrine also participates in the collateral development of mesangial hyperplasia with focal segmental glomerulosclerosis and myocardial mediolysis and apoptosis in subjects with markedly elevated heart rates. *Conclusion*. Norepinephrine coupling with plastically elevated alpha-1 adrenoceptor or other pressor agents generates SAM, a histologically recognizable vasospastic arteriopathy, that with repair is transformed into several different standardized arterial diseases that alter SAM's clinical profile from a hemorrhagic to an ischemic disorder.

## 1. Introduction

An investigation into the genesis of segmental arterial mediolysis (SAM), an uncommon arteriopathy causing potentially catastrophic abdominal, retroperitoneal, and basilar subarachnoid hemorrhages, uncovered a pathogenesis caused by norepinephrine having a distinctive morphology. This article will review this hypothesized pathogenesis, its histology, and generated clinical presentations and show how SAM additionally created and/or contributed to the genesis of other arterial disorders having multiple etiologies. Analyzed also are the vascular lesions generated by the crosstalk of norepinephrine with other pressor agents and the genesis of the collateral myocardial and glomerular lesions that occasionally accompany SAM.

## 2. Materials and Method

The figures exhibited in this article were chosen from multiple cases of SAM reported by the author and coworkers or from cases reviewed in consultation. Their journal origins are listed with the photographic descriptions. The figures were selected to compliment the text by visualizing SAM's multiple evolutionary pathways and its crosstalk and collateral lesions. Indeed, the putative biochemical responses generating SAM have been deduced from these photographs. Moreover, since SAM is rare, its evolutionary pathology is unfamiliar to many physicians, a condition remedied by the multiple photographs that illustrate the full range of SAM's histologic presentations.

### 2.1. SAM Pathogenesis

**Figure 1:**
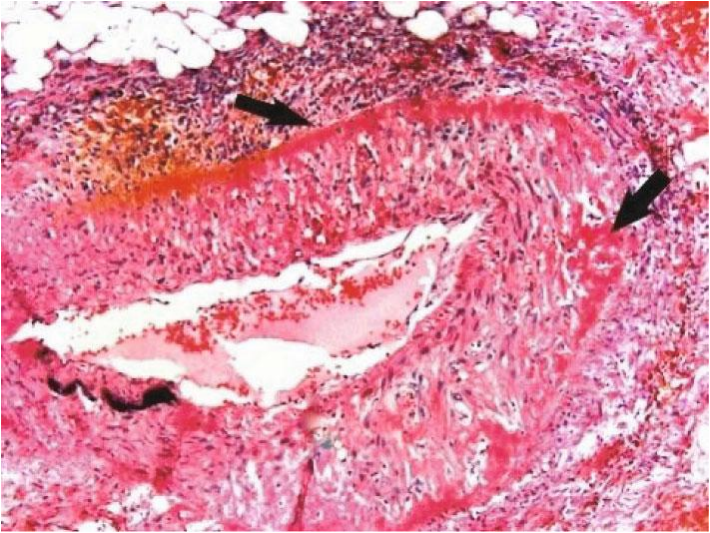
Segmental arterial mediolysis injurious phase with a vasospastic appearance. The top arrow points to the adventitial medial junction circumscribed by a thick linear band of fibrin that at lower arrow extends into and focally fills and obscures areas of mediolysis of the outer half of the media. Hematoxylin & eosin ×50 (adapted from the WJCD 2013.3 published online).

**Figure 2:**
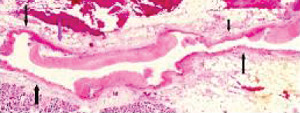
Segmental arterial mediolysis injurious phase. Longitudinal segment of pancreatic artery with arrows pointing to segmentally formed arterial gaps. The magenta arrow points to a beginning dissection that will ultimately either detach the muscular island to enlarge the gap or create a dissecting aneurysm. Hematoxylin & eosin ×10 (adapted with permission from Laboratory Investigation 15(1) copyright 1976, Nature Publishing Group).

**Figure 3:**
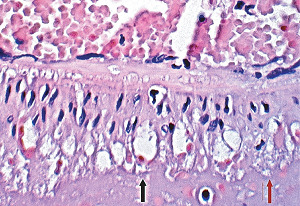
Segmental arterial mediolysis injurious phase. Initial phase of mediolysis. The black arrow points to large, and the red arrow to smaller cytoplasmic vacuoles containing nonstaining clear contents (water) distributed in the outer medial muscle. Hematoxylin & eosin ×400 (adapted with permission from Veterinary Pathology 52(6), copyright 2015, Sage Publications Inc.).

SAM was initially entitled “Segmental Mediolytic Arteritis” because it was suspected of being an immune complex vasculitis [1]. However, an examination of additional cases revealed that this hypothesis was incorrect [2, 3] because SAM was generally unaccompanied by inflammation, failed to display concomitantly occurring laboratory evidence of specific immunologic perturbations, and was often very localized to age-related specific sites. This necessitated changing its name and its supposed inflammatory pathogenesis. A new title, SAM, was coined based solely on its most striking morphologic characteristics and a fresh pathogenesis proposed stating that SAM was a vasospastic arteriopathy stimulated by norepinephrine [4–6]. This credulous hypothesis was formulated from the following salient facts:

1. The restriction of SAM to large- and medium-sized muscular arteries innervated by the peripheral sympathetic nervous system
2. SAM's vasospastic appearance (Figure 1) and segmental spastic distribution (Figure 2) with initial lesions in the outer media (Figure 3) adjacent to the location of the norepinephrine liberating varicosities located on the innervating efferent sympathetic nerve fibers
3. SAM's rapid development, an inherent characteristic of the autonomic nervous system, following the iatrogenic administration of alpha-1 adrenoreceptor agonists and beta-2 agonists capable of releasing norepinephrine from the peripheral sympathetic nervous system [6]
4. SAM's occurrence in clinical conditions of extreme stress that could provoke the release of supraphysiologic levels of norepinephrine from the adrenal medulla [7]
5. The occurrence of renal artery lesions in patients with pheochromocytomas identical to those created in reparative-phase SAM [8]

### 2.2. Injurious-Phase SAM

**Figure 4:**
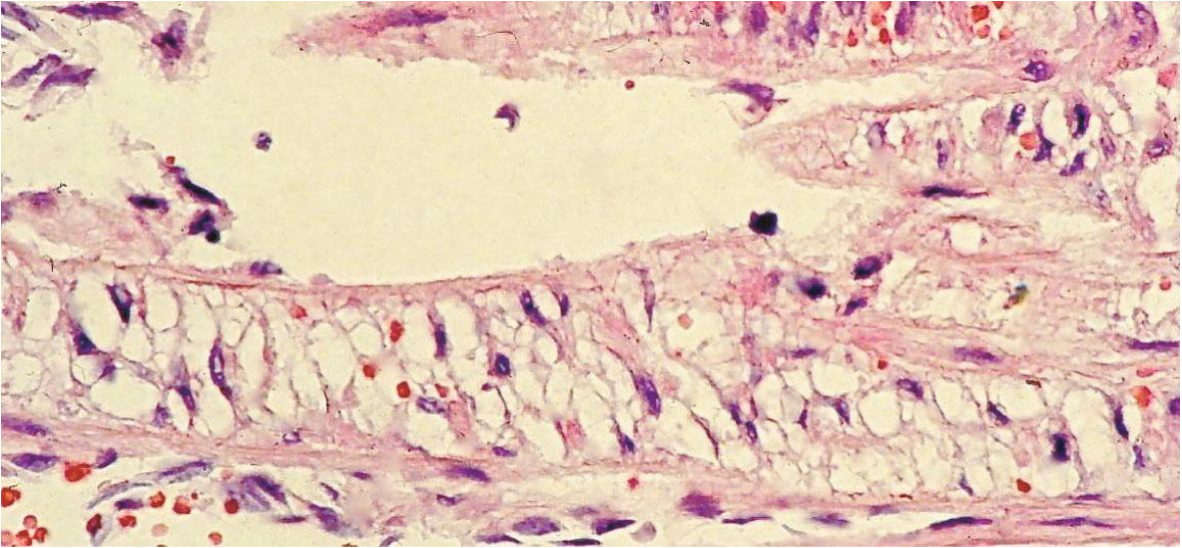
Segmental arterial mediolysis, injurious phase. The arrow points to a longitudinal section of the coronary artery exhibiting transmural cytoplasmic hydropic change. The cell membranes of many scattered swollen myocytes are intact although some are disrupted. Hematoxylin & eosin ×200 (adapted from EC Cardiology 4(7) 2918, online publication).

**Figure 5:**
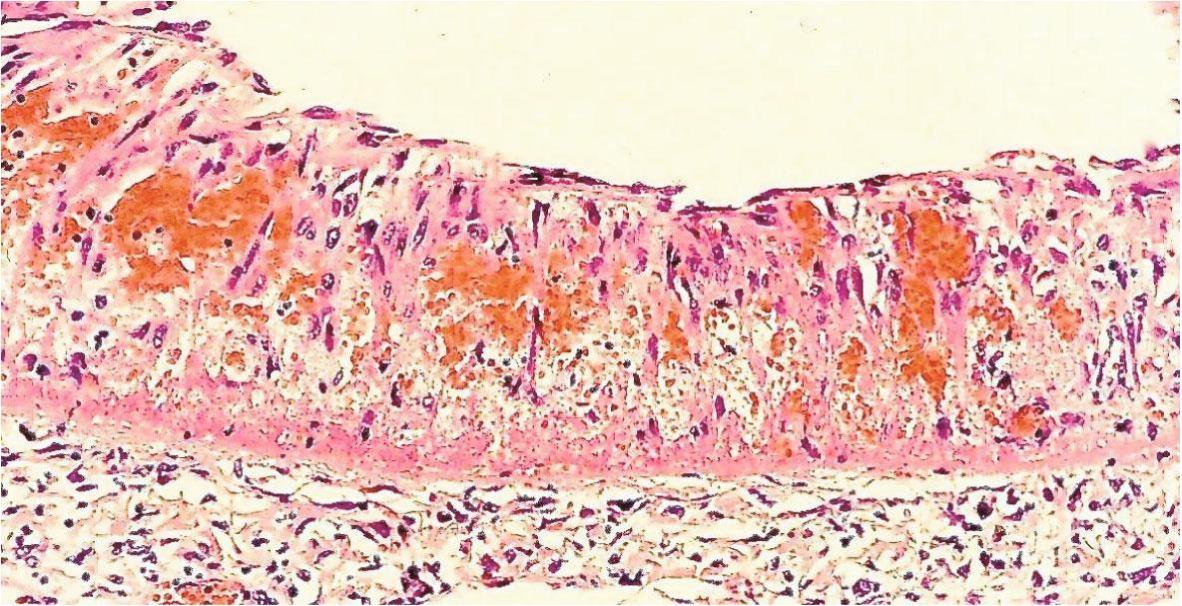
Segmental arterial mediolysis injurious phase. The artery show areas of mediolysis ranging in sites from the outer half to the transmural involvement of the media. Mediolytic foci contain cytoplasmic and membranous debris, scattered viable muscle cells, newly forming capillaries, and red cells. A thick linear layer of fibrin coats the adventitia. The intima is preserved. Hematoxylin & eosin ×200.

**Figure 6:**
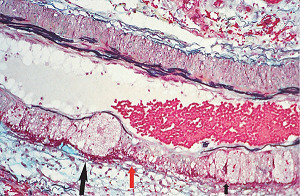
Segmental arterial mediolysis injurious phase. The large black arrow points to a focus of transmural mediolysis with foamy contents that has caused a bulging of the arterial wall bordered inwardly by an intact internal elastica and outwardly by a linear deposit of fibrin. The bulge contents are alcian blue negative. The small black arrow points to a similarly bulging media, and the red arrow to a beginning gap created by the incipient medial loss of the watery mediolytic contents thru an intima depleted of an internal elastica. Movat stain ×100 (adapted with permission from the American Journal of Surgical Pathology 13(7) copyright l989, Lippincott, Williams and Williams).

**Figure 7:**
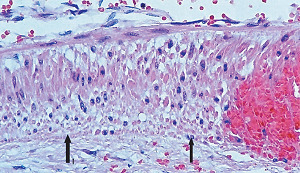
Segmental arterial mediolysis injurious phase. Arrows indicate two foci of apoptosis diffusely involving the outer media with focal extensions into the mid and inner media. A focal microhemorrhage also is evident in the media. Hematoxylin & eosin ×200 (adapted from EC Cardiology 4(7) 2918, online publication).

**Figure 8:**
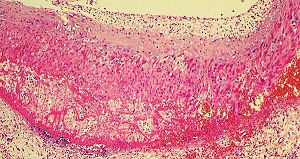
Segmental arterial mediolysis injurious phase. Arrow points to an adventitial-medial tear site outwardly delineated by a linear layer of fibrin. Tear space is edematous and contains fibrin strands and red cells. Fibroblasts and occasional leukocytes are evident in the adjacent adventitial tissue. Hematoxylin & eosin ×100 (adapted with permission from Modern Pathology 8(3) copyright 1995, Nature Publishing Group).

**Figure 9:**
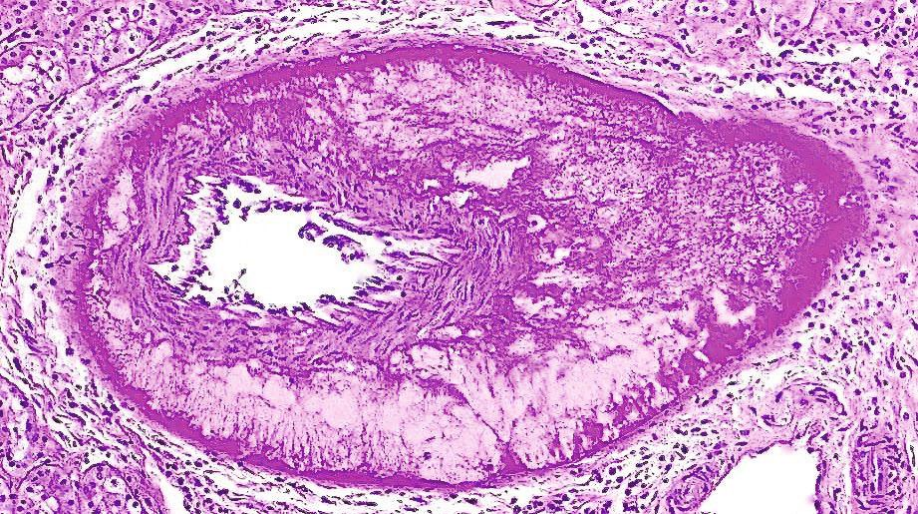
Segmental arterial mediolysis injurious phase. Pig renal artery showing a circumferential separation of the outer media from the adventitia. An edematous tear space containing fibrin strands outwardly pushes the adventitia away from the media. PAS stain ×100 (adapted with permission from Veterinary Pathology 52(6), copyright 2015, Sage Publications Inc.).

The stimuli initiating SAM are produced by iatrogenic sympathomimetic agonists or endogenous supraphysiological levels of catecholamines capable of causing the release of excessive norepinephrine from varicosities on the efferent branches of the peripheral sympathetic nerves. The liberated norepinephrine couples to zones of hyperdense alpha-1 adrenoceptor distributed on the cell membranes of medial smooth muscle [9]. It is this hyperdensity that converts vasoconstriction to vasospasm and is generated by this receptor's dynamic state a plasticity influenced by a variety of exogenous and endogenous factors such as age, sex, and prior exposure to appropriate iatrogenic agonists all important states or conditions encountered in the various clinical presentations of SAM [10, 11]. The normal genetically determined positions of this receptor can be overridden by these factors to create zones of hyperdensity. Not yet determined is whether all or only subdivision(s) of the alpha-1 adrenoceptor (alpha-1A, alpha-1B, or alpha-1D) participate in this hyperdensity remodeling. This remodeling occurs in ages ranging from the fetus to the elderly. Conformational changes in the hyperdense adrenoceptor ligand excessively activate the heterotrimeric Gq protein in the cytoplasm of the medial muscle to unleash an exaggerated biochemical response causing a powerful muscular contraction that shears the arterial media from the adventitia and a cytoplasmic overload of calcium ions released from the sarcoplasmic reticulum and by an increased or prolonged entrance of this ion through calcium channels on the sarcolemma of the smooth muscle cell powered by the activation of phospholipase C and IP3 (triphosphoinositol). These two events create the lesions of injurious-phase SAM. The cytoplasmic calcium ion overload is toxic to mitochondria creating organelle dysfunction that signals the excessive step wise reduction of O_2_ to water by reactive oxygen species (ROS) and an interference with the mitochondrial permeability transition pore [12]. The altered mitochondria become hydropic and dilate (Figure 3). The release of water from multiple injured mitochondria floods the cytoplasm with water creating large intracellular nonstainable distended cytoplasmic vacuoles, some containing membranous residues (Figure 4). These vacuoles, first forming in muscle cells located in the outer media, become confluent through cell membrane disruption. The expanded watery cytoplasm of the coalesced muscle cells has a foamy appearance created by scattered membranous and organelle debris (Figure 5). The added cytoplasmic water floods into the adjacent interstitial tissue distending the adventitial and/or intimal medial boundaries (Figure 6). Mediolysis is the histologic end result of this process. Another consequence of the mitochondrial injury is the release of cytochrome C into the cytoplasm from the inner mitochondrial membrane where it signals a caspase cascade resulting in apoptosis (Figure 7). This event explains how both mediolysis and/or apoptosis can exist in injurious-phase SAM, each process putatively determined by the toxic calcium ion concentration in the cytoplasm enveloping each injured mitochondrion. The other lesion generated in injurious-phase SAM is provoked by an overvigorous vasoconstriction. This shears the outer media away from the external elastica and adventitia causing fibrin deposition along the plane of the tear and within the media (Figure 1). This is frequently associated with microhemorrhages and occasional liberated leukocytes that are distributed around the adventitial medial border and within the media and is derived from torn capillaries and vasa vasorum (Figure 8). More intense vasoconstriction detaches stromal medial adventitial connections causing significant single, multiple, or circumferential separations of the outer media from the adventitia (Figure 9). Arteries showing this change may also exhibit mediolysis although the two injurious-type lesions may separately occur. Zones of mediolysis always involve the outer media but frequently extend into the mid media or are transmedial (Figure 5). Varying widths of mediolysis can develop in different arteries or in different areas of the same artery. The outer medial muscle cells always participate since these muscles directly receive sympathetic innervation whereas the mid and inner medial muscle cells are exposed to norepinephrine by diffusion. Thus, the presence or absence of mediolysis or apoptosis depends on the relative strength of the sympathetic discharge and the breadth of muscle cells. Detected in areas of mediolysis or apoptosis are scattered viable muscle cells, putatively spared because of the nonhomogeneous hyperdense remodeling of alpha-1 adrenoceptor on the medial muscle cells.

**Figure 10:**
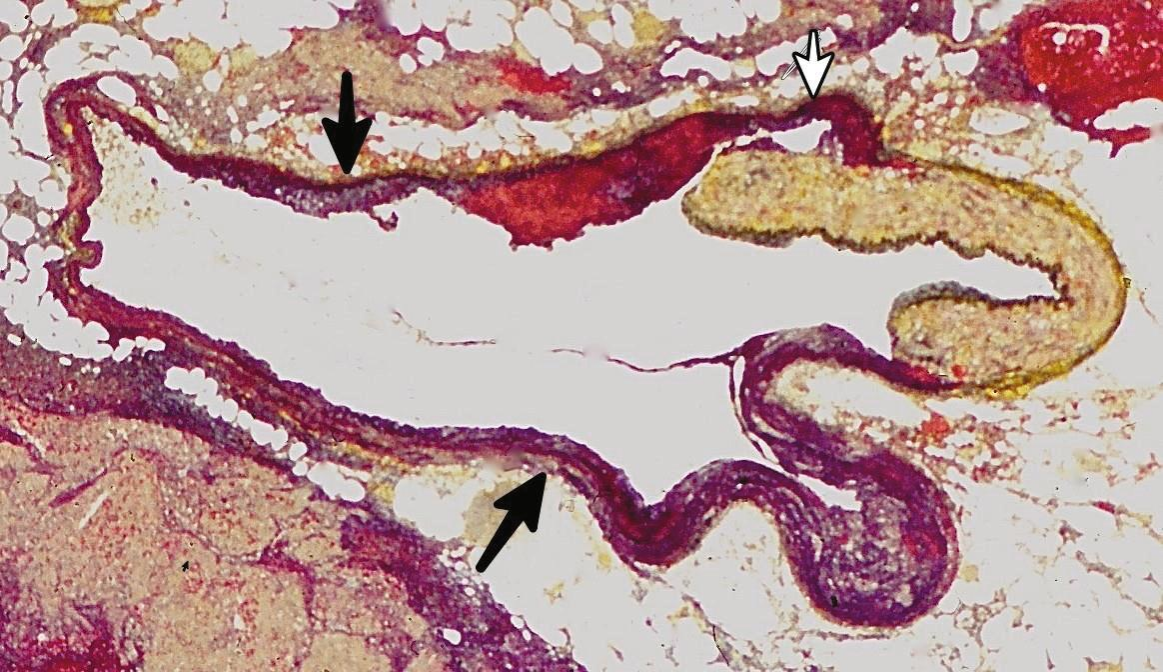
Segmental arterial mediolysis injurious phase. The black arrows point to a large fibrin outlined gap aneurysm. The white arrow shows the site of an early dissection. Movat stain ×25 (adapted with permission from Cardiovascular Pathology 18(6) copyright 2009, Elsevier Inc.).

**Figure 11:**
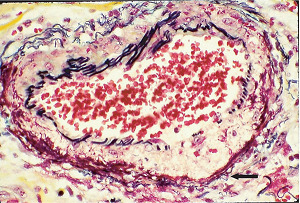
Segmental arterial mediolysis injurious phase. The arrow points to a segment of the artery showing transmural mediolysis with patchy loss of the internal elastica and intima alterations that will allow the watery medial contents to be evacuated into the blood stream creating the gaps illustrated in Figures 12 and 13. The expanded watery mediolytic contents have pushed the fibrin-lined adventitia outwards. Movat stain ×50 (adapted with permission from the American Journal of Surgical Pathology 13(7) copyright l989, Lippincott, Williams and Williams).

**Figure 12:**
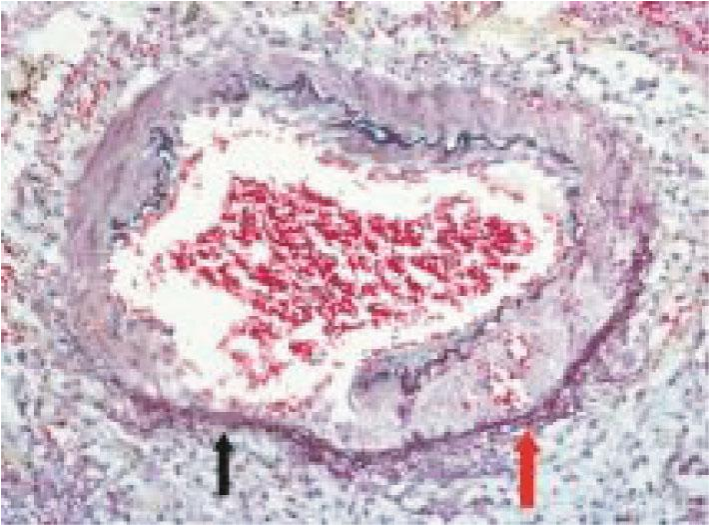
Segmental arterial mediolysis injurious phase. The black arrow points to an arterial gap lined by a linear adventitial fibrin deposit. The adjacent arterial wall at the red arrow shows total medial replacement with granulation tissue consisting of a few dilated capillaries in expanded extracellular matrix material. The internal elastica here is preserved. Movat stain ×50 (adapted with permission from the American Journal of Surgical Pathology 13(7) copyright l989, Lippincott, Williams and Williams).

**Figure 13:**
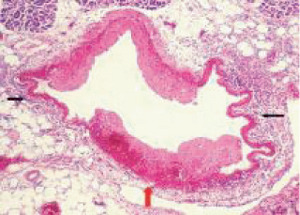
Segmental arterial mediolysis injurious phase. The red arrow points to a medial island, situated between two fibrin-lined gaps (black arrows), showing early detachment provoked by hemorrhage from injured granulation tissue in the outer media and adventitial-medial junction. Hematoxylin & eosin ×100 (adapted with permission from the International Journal of Surgical Pathology 15(2) copyright 200, Sage Publications, Inc.).

The cause of the dramatic hemorrhages announcing injurious-phase SAM is gap aneurysms (Figure 10). These develop from arterial gaps created in areas of transmedial mediolysis and concurrent loss of the internal elastica and intima thru the disruption of muscular stromal connections (Figures 11 and 12). Large gaps will dilate to form aneurysms, but these often develop when segments of uninvolved arterial wall and medial islands, distributed between smaller gaps, become detached from the adventitia by dissections beginning at the interface of the gap with the intact arterial medial island wall and/or by microhemorrhages at the adventitial medial junction and/or media caused by the injury of the granulation tissue capillaries (Figure 13). Medial island detachment in the elderly is promoted by aging degenerative changes of stromal adventitial connections [13].

### 2.3. Reparative-Phase SAM

**Figure 14:**
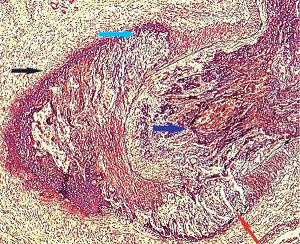
Segmental arterial mediolysis reparative phase. Early reparative change in media and intima. The red arrow points to young granulation tissue filling and expanding a focus of transmural mediolysis and extending thru a fragmented internal elastica to form a broad intimal plaque severely narrowing an arterial lumen (blue arrow) already compromised by a recent thrombus. The black arrow points to a markedly outwardly expanded fibrin-lined adventitia outlining an edematous fibrin stranded zone of mediolysis that shows at light blue arrow another focus of granulation tissue. Movat stain ×100 (adapted from the Journal of Cardiovascular Disease and Diagnosis 2015.32, online publication).

**Figure 15:**
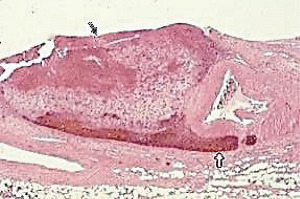
Segmental arterial mediolysis reparative phase. Early reparative phase. The black arrow points to a florid proliferation of young granulation tissue developing in the tear space between the outer media and adventitia. Bleeding from injured capillaries has initiated hematoma formation. The white arrow points to a beginning dissection between the outer media and adventitia. Hematoxylin & eosin ×15 (adapted with permission from the International Journal of Surgical Pathology 15(2) copyright 2007, Sage Publications, Inc.).

**Figure 16:**
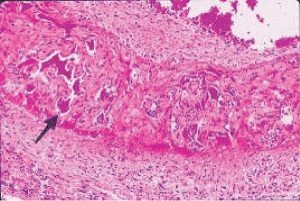
Segmental arterial mediolysis reparative phase. The arrow points to a large irregularly dilated capillary in young granulation tissue repairing a focus of trans medial mediolysis. Hematoxylin & eosin ×200 (adapted with permission from Cardiovascular Pathology 18(6) copyright 2009, Elsevier Inc.).

Norepinephrine also incites the robust development of granulation tissue created to repair the injurious-phase lesions (Figures 14 and 15). Because it is generated very rapidly, often concurrently with evolving injurious-phase lesions, the term chronic to characterize this phase was not appropriate although the resultant repair lesions could be identified weeks to years after the onset of SAM. Therefore, the more general title reparative phase was coined to encompass all the time frames in this phase of SAM's development. The appearance of the granulation tissue echoes these varying developmental time frames. Granulation tissue, formed soon after the onset of SAM, contains multiple irregularly dilated capillaries, occasionally forming angiomatoid-like lesions (Figure 16). Their injury is the cause of the hemorrhages found in the media in the injurious phase (Figures 5 and 7) and the hematomas and dissecting hematomas occurring in reparative-phase sequelae. Mature granulation tissue, viewed in later evolutionary intervals, is primarily composed of extracellular matrix material with few discernable capillaries. This repair creates several sequelae that transform SAM into a multiguised arteriopathy having a different clinical profile. Their differing appearances are determined by the breadth of lesions created in the injurious phase and the extent of their repair. The following arterial lesions are generated.

### 2.4. Arteriopathy with Nonspecific Focal Medial and Adventitial Fibrosis

**Figure 17:**
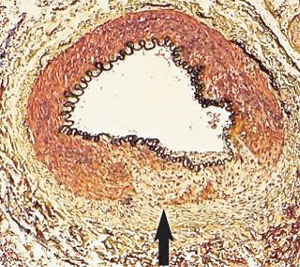
Segmental arterial mediolysis reparative phase. The arrow points to foci of reparative granulation tissue undergoing early fibrosis (yellow stain) in the media and in the adventitia appearing as a crescent that partially encircles the artery. The latter putatively represents an antecedent to adventitial fibromuscular dysplasia. Movat stain ×100 (adapted with permission from Cardiovascular Pathology 18(6) copyright 2009, Elsevier Inc.).

These reparative lesions often accompany the sequelae, described below. They form only when the internal elastic lamina and intima are preserved in injured foci of mediolysis (Figure 17). This repair may develop in asymptomatic but angiographically recognizable SAM lesions and is responsible for their angiographic resolution in follow-up studies [14]. These findings and the serendipity discovered cases of SAM strongly hint that injurious-phase SAM also has subclinical presentations and may be a more common disease than suggested by literature reports [15] chiefly occurring in apparently disease-free children and premenopausal women.

### 2.5. Arteriopathy with Single or Multiple Aneurysms

**Figure 18:**
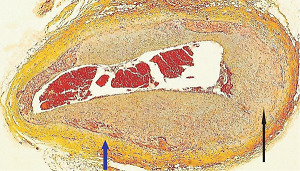
Segmental arterial mediolysis reparative phase. The black arrow points to thick mature granulation tissue filling a large gap aneurysm. The blue arrow shows extended intimal plaque bedecking a medial island that show foci of medial loss with reparative fibrosis. Another granulation tissue gap repair is evident in the upper portion of the artery, Movat stain ×25.

These aneurysms stem from the florid granulation tissue repair of arterial gaps and unruptured gap aneurysms. They are outlined by thick plaques of granulation tissue that often extend beyond the aneurysm to bedeck adjacent medial islands and the intima of adjoining normal arterial segments (Figure 18). Repaired segmentally distributed gaps and large gap aneurysms create large fusiform-shaped aneurysms. Their walls may be further fortified by organizing thrombi. These aneurysms can persist and remain unchanged in follow-up studies [15].

### 2.6. Arteriopathies Creating Vascular Stenosis

Repaired SAM generates three different types of lesions causing arterial stenosis. These transform SAM from a disorder presenting with focal massive hemorrhages to a disease characterized by local or systemic ischemic manifestations.

**Figure 19:**
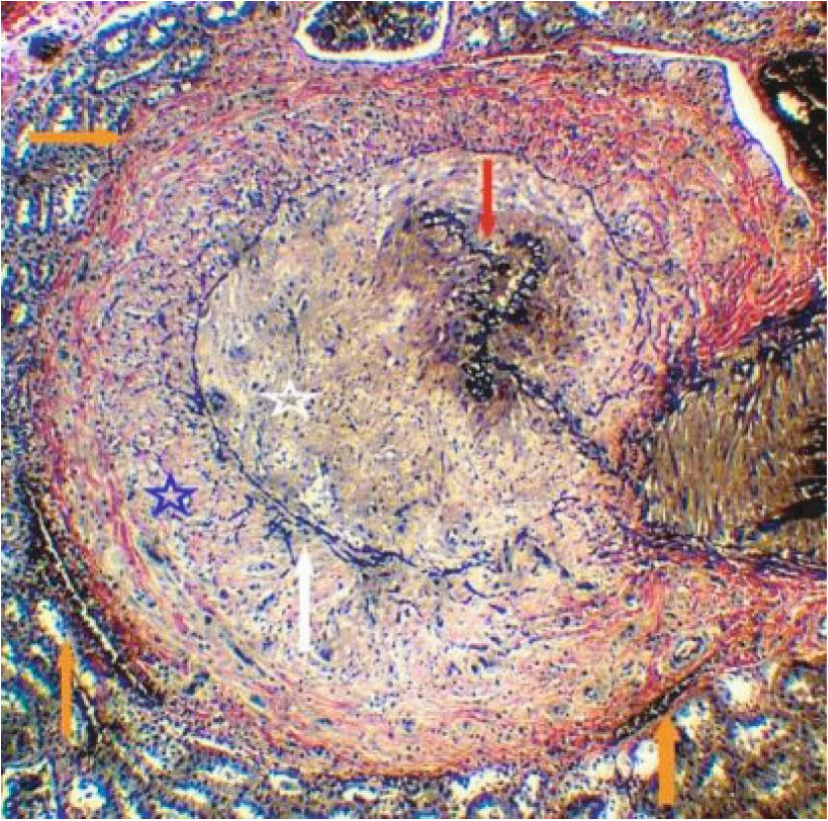
Segmental arterial mediolysis reparative phase. The renal artery lumen identified by the internal elastica at the red arrow is severely compressed by a circumferential proliferation of granulation tissue replacing the outer 2/3 of the media (white star) and the lower and left mid adventitial tissue (blue star). The white arrow points to the external elastica (black membrane), and the red arrows point to the adventitial tissue. Movat stain ×100 (ad4pted with permission from Veterinary Pathology 52(6), copyright 2015, Sage Publications Inc.).

The first stenotic lesion is generated by a florid granulation tissue proliferation in zones of extensive mediolysis. If the internal elastic lamella and intima are preserved, this will compress the arterial lumen (Figure 19), but if the internal elastica is fragmented, the granulation tissue will extend through its defects to generate a stenosing intimal plaque (Figure 14). The arterial lumen can be further narrowed by thrombi.

### 2.7. Dissecting Hematomas and/or Aneurysms

**Figure 20:**
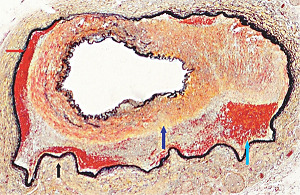
Segmental arterial mediolysis, reparative phase. The cyan arrow points to a hematoma formed in the mature granulation tissue filling and expanding the adventitial medial tear. Adjacent scattered red cells are gathered at the external elastica (black arrow). The red arrow points to a large dissecting hematoma bordered by an intact external elastica. Part of this dissections is evident in the superior view of the artery, and a beginning dissection appears to be generating near the black arrow. The blue arrow points to foci of outer medial fibrosis (yellow stain). Movat stain ×25.

**Figure 21:**
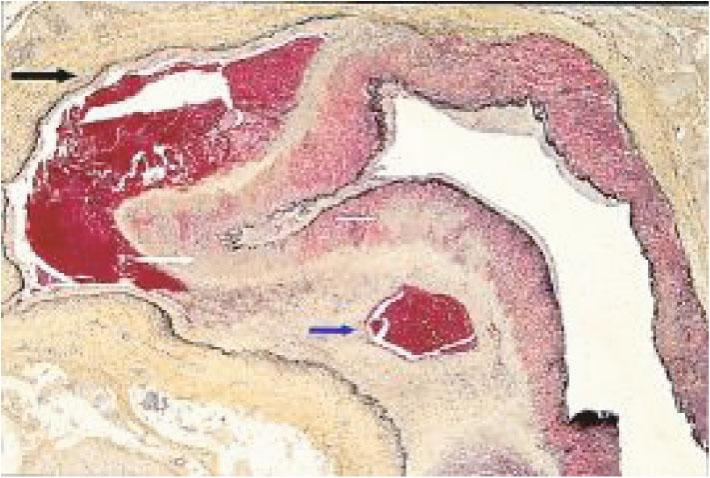
Segmental arterial mediolysis reparative phase. The tear space between the outer media and adventitia is widely expanded by mature granulation tissue containing a hematoma (blue arrow) and a dissecting aneurysm (black arrow). The white arrows point to foci of mature granulation tissue in the outer media a clue to SAM's genesis of the dissection. Movat stain ×15 (adapted with permission from the International Journal of Surgical Pathology 15(2) copyright 2007, Sage Publications).

**Figure 22:**
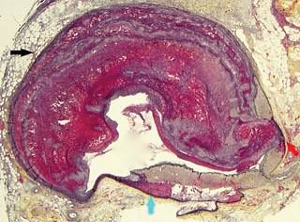
Segmental arterial mediolysis reparative phase. The black arrow points to a gap aneurysm containing a recent thrombus, and the blue arrow to a dissection beginning at the interface of the gap and the arterial wall and developing between the outer media and the fibrin-coated adventitia. Movat stain ×50 (adapted with permission from Modern Pathology 18(3), copyright 1995, Nature Publishing Group).

Dissecting hematomas and/or aneurysms are the second lesions producing arterial stenosis. These always develop in the tear space between the outer media and adventitia (Figures 15, 20, and 21). The hematomas are generated from bleeding from apparently injured granulation tissue capillaries, and this blood may further track to the adventitia and flow along this guard rail to create dissecting hematomas bordered by intact adventitia (Figure 20). Dissecting aneurysms created in SAM always begin between the outer media and adventitia of an intact segment of the arterial wall at its interface with an arterial gap or gap aneurysm (Figure 22). These lesions generally appear in the early reparative phase but can be identified weeks, months, and as late as two years after the onset of SAM. Moreover, they may be provoked by the faulty placement of stents into stenosed arteries [14].

### 2.8. Arterial Fibromuscular Dysplasia (FMD)

**Figure 23:**
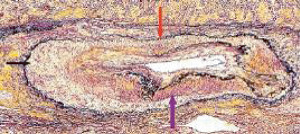
Segmental arterial mediolysis reparative phase showing evolution to FMD. The black arrow points to expanded tear space bordered by an intact external elastica filled with granulation tissue that extends upwardly at the red arrow to totally replace an irregular zone of lost medial muscle. The upper left internal elastica is missing, and the lumen is narrowed by a circumferential plaque of granulation tissue. The purple arrow points to a zone of intact media showing focal fibrosis. Movat stain ×25 (adapted with permission from Cardiovascular Pathology 18(6) copyright 2009 Elsevier Inc.).

**Figure 24:**
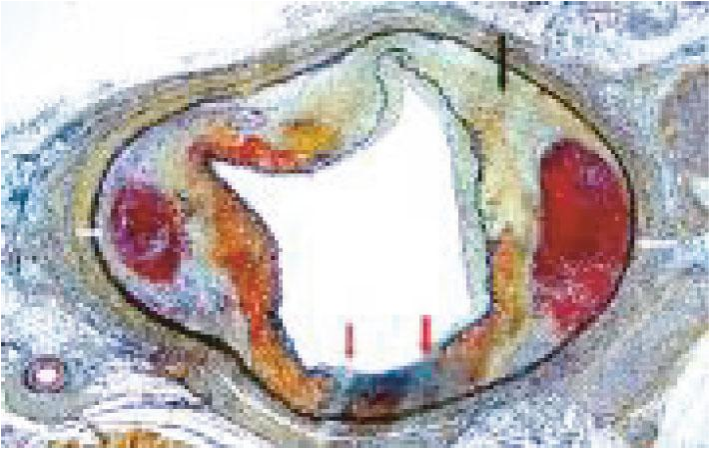
Segmental arterial mediolysis reparative phase evolved to FMD with intramural hematomas. The black arrow points to a large zone of granulation tissue expanding the tear space and reoccupying areas of lost media. The white arrows point to two hematomas generated in the granulation tissue. The red arrows point to two foci of transmedial granulation tissue bordering a central medial island. Movat stain ×25 (adapted with permission from Cardiovascular Pathology 18(6) copyright 2009, Elsevier Inc.).

Arterial fibromuscular dysplasia (FMD) is the third stenotic lesion generated from SAM. The type of FMD formed depends upon the breadth of the injurious-phase lesions. Fibrosis of granulation tissue principally distributed in wide adventitial-medial tear spaces will create perimedial FMD. Periadventitial FMD is fashioned from an adventitial granulation tissue response to vasospasm (Figures 17), and medial FMD, the classic version of this entity, is generated from the granulation tissue repair of both medial and tear space lesions with stenosing intimal plaques forming over zones of fragmented or lost internal elastica (Figures 23 and 24). The above reparative lesions may exist solely as distinct entities or as components of FMD. For example, Figures 20 and 21 can be construed as hematomas and aneurysms developing in perimedial FMD and Figure 24 shows hematomas in medial FMD. Occasionally, metaplastic nodules of smooth muscle may be generated in adventitial repair sites a process suspected of being related to the development of leiomyomas in female rodents administered ractopamine [16].

### 2.9. General Vascular Presentations

**Figure 25:**
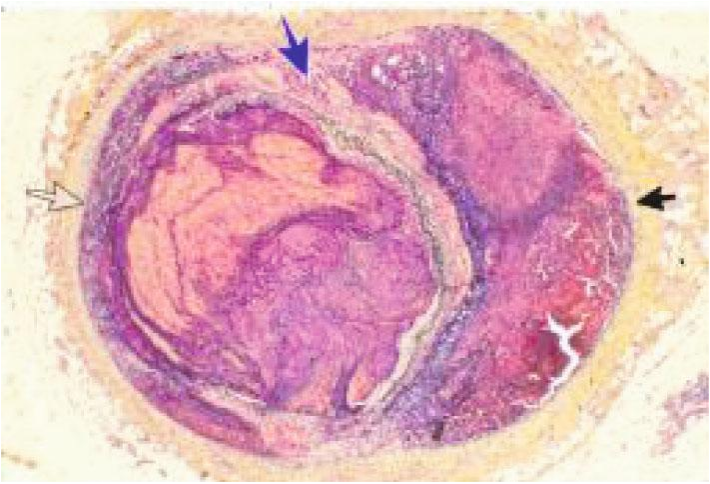
Segmental arterial mediolysis. Cross section of an artery with a double aneurysm. The black arrow points to a dissecting aneurysm, the clear arrow to a gap aneurysm, and the blue arrow to arterial wall segment. Movat stain ×25 (adapted with permission from Cardiovascular Pathology 18(6) copyright 2009, Elsevier Inc.).

SAM is a nonsystemic arteriopathy. It only occurs in the large- and medium-sized muscular arteries located in the abdomen, retroperitoneum, heart, and brain base. SAM's presentation generally is restricted to one of these anatomic sites, although simultaneous occurring SAM in more than one site have been reported most frequently in intestinal and retroperitoneal arteries [15]. The patient's age and sex play a role in SAM's varying clinical profiles. Cardiac symptoms, principally generated from reparative-phase arterial lesions, are restricted to young patients ranging in age from the fetus to young adults. Hemorrhages caused by ruptured gap aneurysms usually only develop in late middle-aged to elderly patients with no sexual preference, while ischemic symptoms produced from reparative sequelae mainly occur in children or young to middle-aged females, data chiefly derived from the clinical profiles of patients with FMD, spontaneous coronary artery, and vertebral artery dissection. The age and sexual differences in SAM's presentations are caused by the plasticity of the alpha 1-adrenoceptor creating zones of hyperdensity in the elderly of both sexes and in female younger adults [9]. SAM's arterial involvement may be very focal occurring as a single lesion or, because of its segmental character, appearing as multiple adjacently situated lesions. Frequently separate lesions, in different breadths of injurious-phase development, are generated in an involved artery (Figure 5) or in arteries located in the same or adjacent arterial beds. The created lesions may exhibit a unique histologic alteration—a single lesion showing more than one type of pathologic change, the most egregious example represented by SAM's double arterial aneurysm (Figure 25).

SAM can develop in all the large abdominal muscular arteries but most frequently targets the celiac axis and its two major branches—the splenic and hepatic arteries [14]. The smallest involved gastrointestinal arteries are located in the submucosa excluding mucosal biopsies as a procedure for diagnosing gastrointestinal SAM. SAM occurs in the distal muscular segment of the renal artery and very focally in one or two of its hilar, lobar, or arcuate branches. Kidney and liver biopsies are often unsuccessful in identifying SAM because of SAM's hilar and focal localizations in these organs. Retroperitoneal organ injury occurs. Large perirenal hematomas are generated from ruptured gap aneurysms of the renal artery or hilar branches and focal infarcts from thrombi in these arteries [15, 17]. Voluminous pancreatic hemorrhages (hemosuccus pancreaticus) develop from ruptured gap aneurysms of the pancreatic branches of the splenic artery [18].

### 2.10. Recurrent SAM

SAM can recur in two tune frames. Early injurious-phase lesions can be superimposed on evolving injurious-phase lesions following administration of sympathomimetic agonists as treatment for hemorrhagic shock [19] or reappear in arteries having reparative lesions, such as FMD, already primed with hyperdense alpha-1 adrenoceptor, following sympathomimetic agonist treatment for other disorders [2].

### 2.11. Multiguised SAM

Granulation tissue repair transforms SAM into several different arteriopathies, each having multiple etiologies. These include the following.

### 2.12. FMD

The cause of FMD is supposedly uncertain. Slavin et al. in 1995 first proposed that SAM was a precursor lesion of FMD [4]. This was contested because of the totally different clinical profiles of both entities, a dilemma reconciled by the recognition of SAM's biphasic clinical profiles [20]. The microphotographs in this article provide uncontestable morphologic evidence showing SAM's transformation into FMD. It also identifies FMD as a standardized reparative response to arterial injury. This view explains FMD appearance in various unrelated conditions causing arterial injury such as trauma and mechanical stress, ischemic episodes, and genetic aberrations. The FMD created from SAM only occurs in arteries innervated by the peripheral nervous system and is created from injurious-phase lesions that are often asymptomatic. This pathogenesis is suspected of being a major cause of intestinal and renal FMD. It most often occurs in young to middle-aged females putatively due to the estrogen generation of foci of hyperdense alpha-1adrenoceptors in the medial muscle. FMD has been reported in patients presenting with spontaneous coronary and vertebral artery dissections. This is not surprising since dissections, as illustrated above, are often constituents of FMD both developing together or as solo lesions. Indeed, in SAM, more than one sequela often is detected in the same artery or in other targeted arterial beds. Intimal FMD, an entity reported in the arteries of the extremities of children, does not develop from SAM [21]. However, intimal lesions do occur in SAM (Figures 14 and 23) but these either are generated by medial granulation tissue extending thru a fragmented or lost internal elastica or by organized thrombi.

### 2.13. Spontaneous Coronary Artery Dissection (SCAD)

SAM is a precursor lesion of SCAD. The following features support this proposal. The targeted arteries in both are the pericardial coronary arteries. The development of SCAD from intimal tears is questionable with dissections and hematomas supposedly arising in the outer media from spontaneous hemorrhage of the vasa vasorum [22]. A more precise etiology of the hematomas arises in SAM from injured granulation tissue in the adventitial medial tear site. Dissections in both entities occur between the outer media and adventitia. FMD in coronary and other arterial systems targeted in SAM have been identified with SCAD, and coronary FMD is considered a likely cause of SCAD [23]. Repaired SAM generates FMD and dissections, and both may be detected in the same artery or arterial bed in SCAD. The clinical profiles of SCAD and coronary artery SAM are similar. Both mainly develop in young patients, young adults in SCAD and infants, children, and young adults in SAM. SCAD and FMD predominantly occur in premenopausal females exposed to stressful conditions including labor and the early postpartum period in SCAD, states that can provoke an excessive endogenous release of norepinephrine from the adrenal gland [24]. Inflammatory SCAD is analyzed in the crosstalk segment.

### 2.14. Vertebral Artery Dissection (VAD)

**Figure 26:**
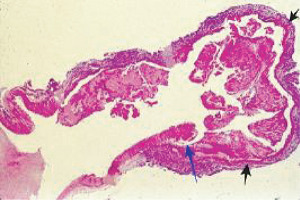
Vertebral artery dissection. The black arrow points to a gap aneurysm and the blue arrow to a beginning dissection. Hematoxylin & eosin ×25.

SAM has been reported as one cause of VAD rendering it another guised entity of SAM [25]. It, like SCAD and FMD, chiefly occurs in children and young or middle-aged adults, appearing at an earlier age in females and developing in arteries free of sclerotic or inflammatory change [26]. It is a leading cause of stroke in young and healthy individuals. VAD is associated with physical and sporting activates and chiropractor manipulations, events that could traumatize or physically stretch the vertebral artery causing intimal damage and tears and/or stimulate the adrenal to release catecholamines [27]. VAD, like SAM, presents with two different clinical profiles but these are anatomically determined—subarachnoid hemorrhages in the intracranial potion of the vertebral, posterior inferior cerebellar and basilar arteries, and ischemic lesions and infarcts developing in the cerebellum, brain stem, and upper spinal cord from stenosis or occlusion of the extracranial segment of this artery and/or its major branches. The two profiles can be explain from etiologic differences of the dissections. Extracranial VAD is hypothesized to be principally derived from intimal tears that generate subintimal hematomas and inner medial muscle bleeding alterations that compress or occlude the arterial lumen. The rapid resorption of the hematoma accounts for the favorable prognosis of this lesion. The subarachnoid hemorrhages are theorized to stem from the transmedial apoptosis of the intracranial segment of the vertebral and/or the posterior inferior cerebellar and basal arteries. The development of this lesion is facilitated by the thin media of the vertebral artery at this site. The apoptosis creates arterial gap aneurysms with dissections, as occurring in SAM, initiated at the gap arterial wall interface with bleeding tracking between the outer media and adventitia (Figure 26). Rupture of this lesion causes subarachnoid hemorrhage with a high rate of rebleeding features responsible for its unfavorable prognosis. SAM can generate this type of intracranial lesion. However, SAM's overall prevalence in the genesis of VAD is uncertain because morphologic studies of this entity are uncommon particularly the extracranial variant that often spontaneously resolves, is successfully treated, or escapes autopsy histologic examination since it is not routinely sought or identified. Moreover, histologic findings in several of the few case reports of intracranial VAD incorrectly diagnose SAM as the cause of the fatal subarachnoid hemorrhages. Clearly, an accurate histologic study is needed to determine the role of SAM in the genesis of VAD particularly since radiologic findings identifying SAM also may not be correct in all cases.

### 2.15. Crosstalk of Norepinephrine with Other Pressor Agents

Three different morphologic presentations of SAM surface in this crosstalk.

### 2.16. Superimposition of SAM on a Preexisting Pressor Response

**Figure 27:**
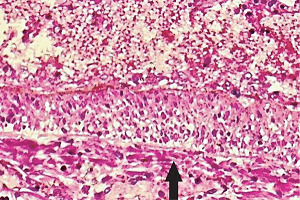
Pulmonary artery segmental arterial mediolysis injurious phase. The arrow points to a large focus of apoptosis involving the outer half of the media. Hematoxylin & eosin ×200 (adapted from Interventional Cardiology 12(3) copyright 2020, online publication).

**Figure 28:**
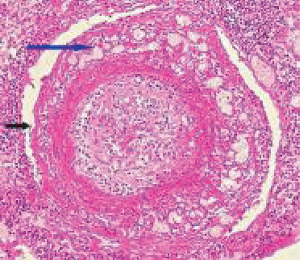
Pulmonary artery segmental arterial mediolysis reparative phase, early. An almost circumferential florid proliferation of young granulation tissue with widely dilated irregularly configured capillaries having an angiomatous appearance (blue arrow) is evident between the adventitia (black arrow) and outer media. The arterial lumen is almost completed filled with a mixed population of endothelial cells, fibroblasts, and myofibroblasts. Hematoxylin & eosin ×100 (adapted from Interventional Cardiology12(3) copyright 2020, online publication).

This was evident in the muscular pulmonary arteries of a 22-year-old black female patient with idiopathic pulmonary hypertension terrorized by a hex [28]. Serotonin (5-HT) is currently considered an important agent in the genesis of idiopathic pulmonary hypertension [29], and terror is a powerful stimulus for the endogenous release of norepinephrine. The lesions of SAM added to those of pulmonary hypertension were zones of medial muscle apoptosis concentrated in the outer media focally progressing to form small arterial gaps and the positioning of young granulation tissue at the adventitial medial interface (Figures 27 and 28). SAM involving the pulmonary arteries is extremely rare despite its innervation by the peripheral sympathetic nervous system and connections of the coronary with pulmonary plexuses. The rarity is putatively attributable to a low density of alpha-1 adrenoceptors in the muscular pulmonary arteries [30]. The summation of biochemical vasoconstricting events generated by the crosstalk of 5-HT and norepinephrine created the calcium ion overload instrumental in focally adding SAM to the histologic alterations of pulmonary hypertension. Infants born with persistent pulmonary hypertension (PPHN) delivered from depressed mothers administered selective serotonin reuptake inhibitors (SSRI) [31] may additionally develop coronary artery SAM by the paracrine crosstalk of stored nonmetabolized pulmonary 5-HT with released norepinephrine in adjoining pericardial arteries.

### 2.17. The Concurrent Development of SAM with Another Pressor Response

Robinowitz et al. reported six cases of SCAD in premenopausal females associated with an eosinophilic adventitial inflammatory response which they suspected was the cause of SCAD [32]. The pathologic descriptions in some cases and those in their literature review were consistent with SAM, an entity devoid of causative inflammation. This incongruity can be resolved by considering this lesion to have been generated thru the crosstalk of two nearly simultaneous occurring pressor responses, one caused by norepinephrine and the other by an allergic coronary artery spasm stimulated by histamine. Implicating histamine is the localization of the inflammatory reaction to the arterial adventitia, a home site of mast cells, the producers of histamine and an inflammatory response composed chiefly of eosinophils, cells recruited to sites of histamine production by their H4 histamine seeking receptor [33]. This double pressor response may have been precipitated by a concurrent allergic response to the sympathomimetic agent initiating SAM or by the medial hyperreactivity caused by norepinephrine signaling, an allergic pressor response [34]. The summation of both pressor responses would create the calcium ion overload generating SAM. The toxic proteins in the eosinophil's granules may have further injured the artery by disrupting the capillaries in the reparative granulation tissue formed in the outer media and adventitial-medial tear, to create the dissecting hematomas of SCAD.

### 2.18. SAM and Accompanying Venous Angiopathy Forming Venous FMD

**Figure 29:**
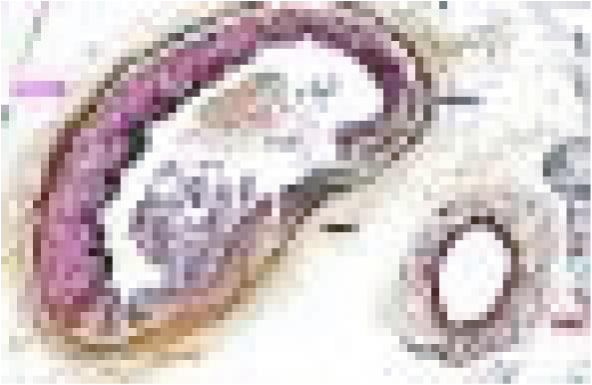
Vasospastic venous angiopathy. The red arrow points to a vein with irregular outer contours thickened by reparative alterations—edema, granulation tissue, and patchy loss of muscle in the adventitia. The adjacent artery (purple arrow) shows SAM induced change—transmedial granulation tissue (orange arrow), gap (black arrow), and a dissecting hematoma (blue arrow). Movat stain ×25 (adapted from the World Journal Cardiovascular Diseases 2013.3 published online).

**Figure 30:**
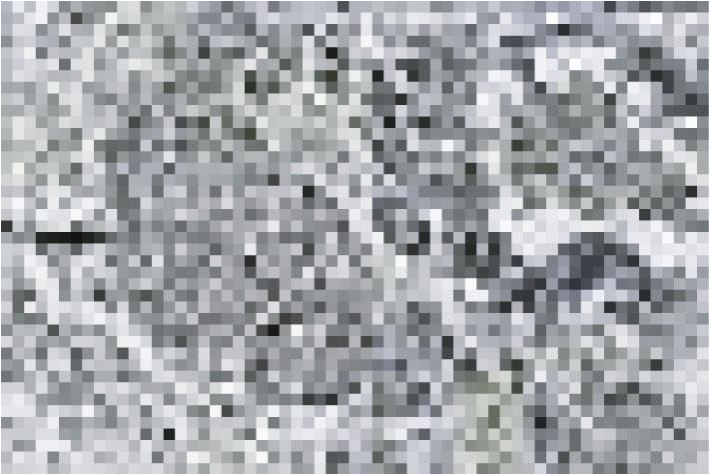
Vasospastic venous angiopathy. The black arrow points to a focus of adventitial muscle showing hydropic change akin to medial change in Figures 3 and 4. Movat stain ×200 (adapted with permission from the International Journal of Surgical Pathology 15(2) copyright 2007, Sage Publications).

**Figure 31:**
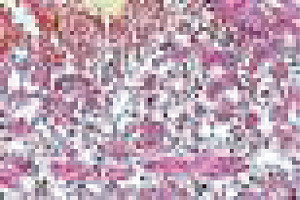
Vasospastic venous angiopathy. Medial muscle showing confluent mediolysis distributed in an edematous background containing membranous and organelle remnants, a few intact muscle cells some with cytoplasmic vacuoles, and scattered apoptotic bodies. Hematoxylin & eosin ×200 (adapted with permission from the International Journal of Surgical Pathology 15(2) copyright 2007, Sage Publication).

**Figure 32:**
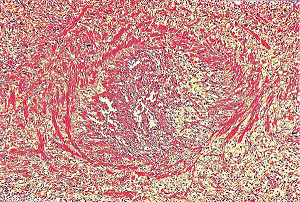
Vasospastic venous angiopathy. Edematous fluid, released from the lysed muscle cells, containing newly forming granulation tissue and cellular debris widely separates surviving medial and adventitial muscle and compresses and disrupts the intima. Hematoxylin & eosin ×100 (adapted from the World Journal Cardiovascular Diseases 2013.3 published online).

**Figure 33:**
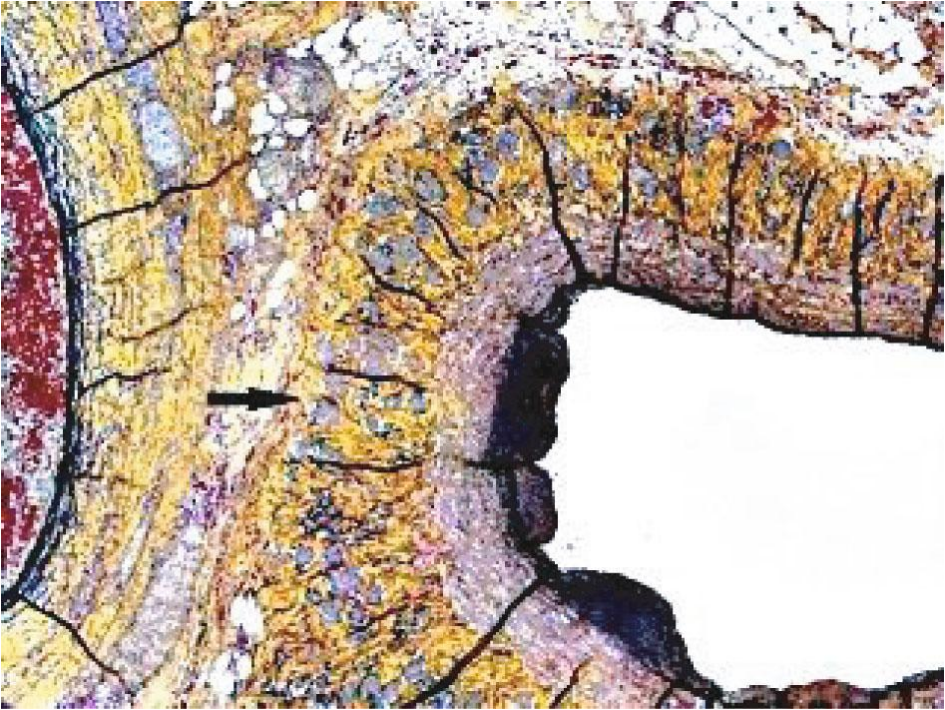
Vasospastic venous angiopathy. The arrow points to renal vein chiefly thickened by fibrosis of the outer media and adventitia that surrounds surviving muscle cells—venous FMD. Movat stain ×50 (adapted from the World Journal Cardiovascular Diseases 2014.4, online publication).

**Figure 34:**
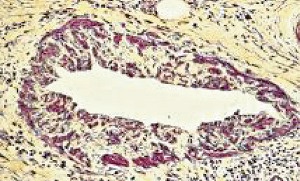
Vasospastic venous angiopathy. The small intestinal vein without significant adventitial muscle shows reparative fibrosis replacing lysed medial muscle providing another morphologic appearance to venous FMD. Movat stain ×100 (adapted with permission from the International Journal of Surgical Pathology 15(2) copyright 2007, Sage Publications).

**A** vasospastic venous angiopathy may closely accompany renal and intestinal artery SAM (Figure 29). It, like injurious-phase SAM, exhibits smooth muscle vacuole development with nonstainable contents (water) (Figure 30), lysis and apoptosis of medial and adventitial muscle (Figure 31), and edema of the media putatively caused by the flooding of the interstitial tissue by water released from the lysing medial muscle (Figure 32). Edematous zones of medial muscle contain membranous and organelle debris and are rapidly reoccupied by loosely created granulation tissue. Its fibrosis creates FMD that like arterial FMD has different histologic profiles (Figures 33 and 34). The loss of adventitial smooth muscle in large veins creates thickened veins exhibiting irregular outer contours while smaller veins with little or no adventitial muscle only exhibit medial muscle loss creating veins with a moth-eaten media [5, 35]. Complicating intimal plaques may form, and thrombi can develop. Immunostaining for endothelin-1 (ET-1) revealed this agent in the venous endothelium and medial muscle's intact and fragmented cell membranes. Positive staining was also evident in the adventitial capillaries of adjacent arteries and veins, in capillaries of adjoining mesenteric fat and in adipose tissue, but ET-1 was absent in the endothelium and medial muscle of the artery afflicted by SAM [5]. This distribution of ET-1 suggests that its release from the vascular endothelium represents a “field effect” putatively stimulated by pathophysiologic effects generated by SAM. ET-1 can cause venous vasoconstriction by forming a ligand with the ETA receptor on the cell membrane of the medial muscle that then couples with a cytoplasmic Cq protein activating a phosphatidylinositol calcium messenger system initiating muscle contraction. However, the venous constriction accompanying SAM is not normal but is vasospastic. This condition is generated by its crosstalk with a norepinephrine-stimulated venoconstriction activated by the same stimulus causing SAM. These two simultaneous developing venous pressor responses create the toxic calcium ion overload responsible for the genesis of this vasospastic lesion. This venous lesion is dissimilar to SAM chiefly because veins do not have an external elastica and smooth muscle also is positioned in the adventitia of large veins so that the sharp shearing of the media from the adventitia found in SAM is absent. The lytic loss of the adventitial muscle and its replacement fibrosis creates the irregular poorly circumscribed adventitia and venous wall thickening that characterize large vein FMD.

### 2.19. Collateral Lesions Accompanying SAM

#### 2.19.1. Mesangial Hyperplasia with Focal Segmental Glomerulosclerosis

**Figure 35:**
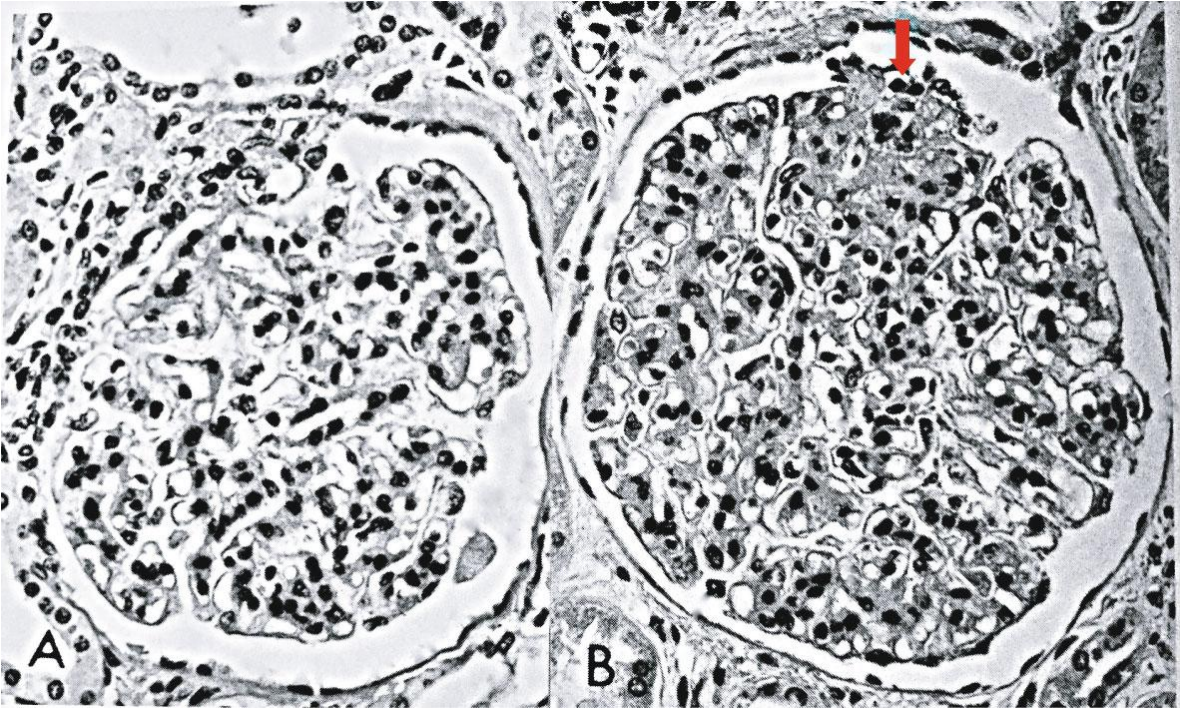
Glomeruli in segmental arterial mediolysis. The glomerulus in (a) exhibits an in tandem arrangement of hyperplastic mesangial cells in the glomerular stalks. Glomerulus in (b) in addition to mesangial hyperplasia also shows at the red arrow a segmental aggregation of matrix material obscuring the adjacent glomerular loop. Hematoxylin & eosin ×400 (adapted with permission from Laboratory Investigation 35(1) copyright 1976, Nature Publishing Group).

Mesangial hyperplasia may coexist with renal artery SAM with norepinephrine also participating in its genesis (Figure 35(a)) [5]. Mesangial cells are myofibroblasts generated from hematopoietic stem cells [36]. The bone marrow is innervated by the peripheral sympathetic nervous system [37] so that the same stimuli that liberate norepinephrine from the varicosities on the efferent branches of the peripheral sympathetic nerves innervating the renal artery may also incite the release of mesangial cells or their precursors from the bone marrow to colonize the mesangial stalk causing their hyperplasia. The mesangial cell besides contributing to central axial glomerular support and as macrophages performing phagocytic functions also regulates glomerular capillary blood flow representing the glomerular homologue to arterial smooth muscle [38]. They possess alpha-1 adrenoceptor, and the biochemical events initiating mesangial cell contraction are similar to those occurring in smooth muscle. The contraction may severely narrow or even obstruct blood flow thru the capillary loops. This response aids the contraction of large renal arteries in diverting blood away from the kidney to other sites for example to striated muscle in a “fight and flight” response, a reaction putatively associated with SAM [7]. The exaggerated biochemical response creating SAM in the large renal arteries initiated by the coupling of norepinephrine with hyperdense foci of alpha-1 adrenoceptor also may occur in these cells causing focal apoptosis with repair promoted by norepinephrine-induced matrix protein production from adjoining intact mesangial cells (Figure 35(b)). These alterations create the morphologic lesions encountered in focal segmental glomerulosclerosis adding another entity to the genesis of this disease. The mesangial hyperplasia is usually focal imitating the locality of SAM's renal arterial involvement.

#### 2.19.2. Myocardial Toxicity

**Figure 36:**
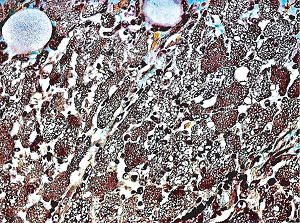
Myocardium in segmental arterial mediolysis. The cytoplasm of most myocytes shows alcian blue-negative vacuoles (hydropic mitochondria). Disruption of the vacuolar membranes has caused the coalescence of their contents filling the cells with minute lakes of water similar to smooth muscle in SAM (Figure 3). Edematous fluid derived from disrupted myocytes containing alcian blue matrix material stemming from the early reparative granulation tissue is evident in the interstitial tissue separating the degenerating myocytes. Movat stain ×200 (adapted from EC Cardiology 5.7 2018, online publication).

**Figure 37:**
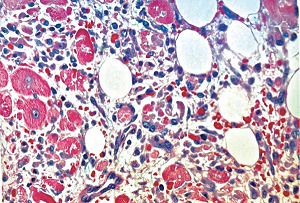
Myocardium in segmental arterial mediolysis. Apoptotic bodies composed of well-circumscribed densely eosinophilic cytoplasmic budding fragments are evident dispersed in newly formed granulation tissue composed of fibroblasts and capillaries. Hematoxylin & eosin ×200 (adapted from EC Cardiology 5.7 2018, online publication).

Identical morphologic changes to those developing in the medial smooth muscle in SAM—apoptosis and hydropic degeneration of the mitochondria—can develop as collateral lesions in the heart muscle in SAM (Figures 36 and 37) [39, 40]. This is rare having been detected in a 28-week-old twin fetus and in two greyhounds [5, 6, 39] both developing shortly after the administration of beta receptor agonists, a tocolytic agent to the mother of the fetus and ractopamine in the dogs. The underlying condition promoting the myocardial toxicity was their markedly elevated heart rates—constant in the fetus and episodic in the greyhounds [40]. An upgraded beta-1 adrenoceptor would be necessary to meet the hemodynamic stress caused by the markedly increased myocardial contractility. This receptor is also dynamically regulated by a variety of pathophysiologic states [41]. An upload may also apply to the alpha-1 adrenoceptor located on the nuclear membrane of the myocytes [42]. The enhanced stimulus for norepinephrine production and release from the sympathetic nerves innervating the myocardium created by the afore mentioned beta agonists would generate an augmented coupling of norepinephrine with the upgraded beta-1 adrenoceptor on the myocyte cell membrane and the alpha-1 adrenoceptor on the nuclear membrane. The ligand generated in the former coupling activates the Gs-cytoplasmic protein or adenylyl cyclase setting into motion an exaggerated calcium ion influx through the L-type calcium ion channel resulting in a cytoplasmic calcium ion overload. This overload may be enhanced by the release of excessive calcium stored in the sarcoplasmic reticulum by the activation of the ryanodine receptor. The ligand created with the alpha-1 adrenoceptor couples with a Gq protein putatively excessively activating phospholipase that cleaves phosphatidylinositol increasing inositol triphosphate (IP3). This binds with the IP3 receptor on the sarcoplasmic reticulum to also release excessive calcium ions. The latter's calcium ion release may be delayed by a latency of the alpha-1 adrenergic response caused by its location on the nuclear membrane. The excessive and prolonged calcium ion release signals ROS in bordering mitochondria to produce the mitochondrial hydrops and lysis and/or the activation of cytochrome C that signals caspase 3 to initiate the caspase cascade ending in apoptosis. A brisk reparative response putatively also instigated by norepinephrine rapidly develops and can accompany the myocardial lesions creating areas of fine fibrosis in the both the right and left hearts.

#### 2.19.3. Diagnostic Histologic Conundrums

**Figure 38:**
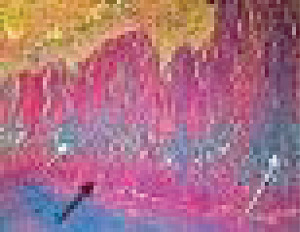
Muscular artery cystic necrosis. The black arrow points to preserved outer media, and the white arrows point to expanded alcian blue-positive extracellular matrix material in mid media. Movat stain ×25 (adapted from the World Journal Cardiovascular Diseases 2013.3 published online).

The gold standard for diagnosing SAM was its histologic identification, but because of treatment options, this was no longer always possible; thus, a second radiologic gold standard was established. Unfortunately, the gold in both options is tarnished since the histologic diagnoses in some reports were incorrect blemishing the accuracy of accompanying radiologic findings and literature reviews of SAM. The certain histologic diagnosis of injurious-phase SAM requires the identification of its two separate distinct lesions—mediolysis or apoptosis and the shearing of media from the adventitia that generates fibrin deposition in the plane of the tear and varying degrees of medial separation from the adventitia. Contributing to this complexity are the edematous expansion of these two lesions, the frequent presence of two or more injurious components in one or adjoining lesion sites, and the rapid imposition of granulation tissue whose capillaries often are irregularly dilated and injured adding hemorrhage and scattered inflammatory cells to both lesions. Apoptosis of the medial smooth muscle is a standardized response to a host of injuries. Therefore, arteriopathies solely exhibiting apoptosis, often accompanied with pools of expanded extracellular matrix material, are not SAM especially if the apoptosis begins in the mid or inner media. Muscular artery cystic necrosis so named because it accompanied elastic arteries exhibiting cystic medial necrosis is an arteriopathy with these morphologic features (Figure 38) identified chiefly in the carotid and iliac arterial beds and most often misdiagnosed as SAM [20]. Its pathogenesis, like its aortic namesake, is uncertain and probably multifactorial. Hemorrhage in the outer media and adventitial medial interface without adventitial fibrin deposition, and absent medial apoptosis likewise is not definitively SAM unless elsewhere accompanied by other morphologic alterations of SAM. Arteriopathies developing arterial gaps not outwardly outlined by fibrin and without evidence of granulation tissue repair are not diagnostic of SAM. Sequelae generated from injurious-phase SAM are not totally specific occurring in other arterial diseases. Clues to pinpoint SAM in their genesis are the histologic identification of persistent and/or transformed alterations acquired in the injurious phase. These include the concomitant appearance of more than one sequela in involved or adjoining arteries or arterial beds, medial islands enveloped by granulation tissue or fibrosis, and foci of medial granulation tissue or fibrosis bordering aneurysms and dissecting aneurysms and hematomas (Figures 18 and 21).

Finally, dissecting aneurysms in SAM always develop between the outer media and adventitia and are initiated at the interface of an arterial gap with intact arterial wall.

#### 2.19.4. Clinical Diagnostic Consideration

Injurious lesions of SAM develop very rapidly and are histologically apparent within a day after exposure to an appropriate stimulus. Therefore, very recent histories of treatment with iatrogenic sympathomimetic agonists, extreme physical exertion, or emotional stress are important clues in establishing a clinical diagnosis of SAM. If the above stimuli are not identified and SAM still suspected, poisoning in meat products with ractopamine should be strongly considered the responsible etiologic stimulus [6]. This drug is a repartition agent used in animal husbandry to stimulate muscle growth and fat loss in pigs, cattle, and turkeys. It is rapidly metabolized. Another suspected agent is clenbuterol. This drug is utilized for weight reduction and by body builders. It also is a beta-2 agonist, having similar pharmacological properties to ractopamine [43]. However, SAM has not been definitively identified in humans or experimental animals given this agent.

## 3. Conclusion

SAM is a rarely reported vasospastic arteriopathy that often causes calamitous hemorrhages in elderly patients. It involves large muscular arteries innervated by the peripheral sympathetic nervous system located in the abdomen, retroperitoneum, pericardium, and posterior brain base. It is also a precursor to standardized cardiovascular and renal disorders having multiple etiologies. These two presentations create a multiguised arteriopathy with two differing clinical profiles, intense hemorrhage or ischemia. SAM, stimulated by iatrogenic sympathomimetic agonists or superphysiological levels of adrenal catecholamines, is initiated by norepinephrine released from varicosities on the efferent sympathetic nerves located in the vicinity of the arterial medial-adventitial junction. A vasospastic response is generated by the coupling of this hormone to hyperdense foci of alpha-1 adrenoceptor. This receptor's dynamic state creates this hyperdensity. The multiple ligands formed couple with the Cq protein to unleash an exaggerated biochemical response generating injurious-phase SAM. Two distinct lesions develop—the shearing of the media from the adventitia and mediolysis and/or apoptosis of medial muscle caused by mitochondrial injury generated by a toxic overload of calcium ions. Medial muscle loss creates arterial gap aneurysms whose rupture causes massive hemorrhages. Norepinephrine also signals a vigorous and rapid reparative response that mends the asymptomatic lesions created in the injurious phase or generates sequelae that add ischemia to SAM's clinical profile. The sequelae are participants in various arterial disorders generated as standardized responses to a variety of arterial injuries. The entities include FMD, SCAD, VAD, and dissecting hematomas and aneurysms of the intestinal, splenic, and renal arteries. Norepinephrine may crosstalk with 5-HT to superimpose SAM on idiopathic pulmonary hypertension and PPHN, with histamine to create and add eosinophils to SCAD and with endothelin-1 to generate a field effect that forms a venous angiopathy terminating as venous FMD. Norepinephrine plays a role in the genesis of collateral lesions that may accompany SAM. It promotes mesangial hyperplasia by liberating mesangial cells from the bone marrow that lodge in the glomerular stalk to cause hyperplasia and incites mesangial cells endowed with hyper dense alpha-1 adrenoceptor to undergo apoptosis with matrix repair generating focal segmental glomerulosclerosis. Beta agonists implicated in the genesis of SAM can cause collateral myocardial apoptosis and mediolysis in situations of hemodynamic stress provoked by increased cardiac contractility. Hyperdense foci of the cardiac B-1 adrenoceptor develop to accommodate this condition. The increased release of norepinephrine stimulated by the beta agonists joined to the hyperdense B-1 adrenoceptor couples with the Gs protein and adenylyl cyclase to excessively activate a series of biochemical responses overloading the cytoplasm with calcium ions causing myocardial hydrops, apoptosis, and rapid repair.
